# Tear proteomics reveals the molecular basis of the efficacy of human recombinant nerve growth factor treatment for Neurotrophic Keratopathy

**DOI:** 10.1038/s41598-022-05229-4

**Published:** 2022-01-24

**Authors:** Damiana Pieragostino, Manuela Lanzini, Ilaria Cicalini, Maria Concetta Cufaro, Verena Damiani, Leonardo Mastropasqua, Vincenzo De Laurenzi, Mario Nubile, Paola Lanuti, Giuseppina Bologna, Luca Agnifili, Piero Del Boccio

**Affiliations:** 1 Center for Advanced Studies and Technology (CAST), University “G. d’Annunzio” of Chieti-Pescara, Chieti, Italy; 2 Department of Innovative Technologies in Medicine and Dentistry, University “G. d’Annunzio” of Chieti-Pescara, Chieti, Italy; 3 Department of Medicine and Aging Science, “G. d’Annunzio” University of Chieti-Pescara, Chieti, Italy; 4 Ophthalmology Clinic, National Centre of High Technology (CNAT) in Ophthalmology, University of “G d’Annunzio” Chieti-Pescara, Chieti, Italy; 5 Department of Pharmacy, University “G. d’Annunzio” of Chieti-Pescara, Chieti, Italy

**Keywords:** Proteomics, Prognostic markers

## Abstract

Neurotrophic Keratopathy (NK), classified as an orphan disease (ORPHA137596), is a rare degenerative corneal disease characterized by epithelial instability and decreased corneal sensitivity caused by the damage to the corneal nerves. The administration of human recombinant nerve growth factor (*rhNGF*) eye drops, as a licensed-in-Europe specific medication for treatment of moderate and severe NK, has added promising perspectives to the management of this disorder by providing a valid alternative to the neurotization surgery. However, few studies have been conducted to the molecular mechanism underlying the response to the treatment. Here, we carried out tears proteomics to highlight the protein expression during pharmacological treatment of NK (Data are available via ProteomeXchange with identifier PXD025408).Our data emphasized a proteome modulation during *rhNGF* treatment related to an increase in DNA synthesis, an activation of both BDNF signal and IL6 receptor. Furthermore, the amount of neuronal Extracellular Vesicles EVs (CD171+) correlated with the EVs carrying IL6R (CD126+) together associated to the inflammatory EVs (CD45+) in tears. Such scenario determined drug response, confirmed by an in vivo confocal microscopy analysis, showing an increase in length, density and number of nerve fiber branches during treatment. In summary, *rhNGF* treatment seems to determine an inflammatory micro-environment, mediated by functionalized EVs, defining the drug response by stimulating protein synthesis and fiber regeneration.

## Introduction

Neurotrophic Keratitis or Neurotrophic Keratopathy (NK) is a rare degenerative corneal disease characterized by epithelial instability and decreased corneal sensitivity caused by damage to the corneal nerves^1,2^. NK is related to an alteration of corneal nerves with consequent impairment in sensory and trophic function, instability of the corneal epithelium, thus influencing the integrity of the tear film, as well as the epithelium and the stroma^1,3^. The hallmark of NK is a decrease or absence of corneal sensation^3^ and the most common causes of this impairment are related to herpetic keratitis, intracranial space-occupying lesions, and/or neurosurgical procedures that damage the trigeminal ophthalmic branch^2–4^. NK is traditionally classified basing on the severity of corneal damage according to the Mackie criteria (2015)^4^. Briefly, stage 1 NK is characterized by corneal epithelial irregularity and tear fluid alteration; in stage 2 NK there are persistent epithelial defects (PED); instead, stage 3 NK is characterized by corneal ulcers with stromal involvement that may lead to corneal melting and perforation^4,5^. Recently, Mastropasqua et al. proposed a new classification of NK implementing the traditional clinical stages with data obtained by corneal diagnostic imaging. In particular, stage 1 and 2 of Mackie classification have been distinguished in 1A and 1B according to nerve fiber density and stage 3 was divided in 3A and 3B according to the percentage of stromal thickness loss^2^. Currently, NK is classified as an orphan disease (ORPHA137596) and was considered a rare disease by the Committee for Orphan Medicinal Products (COMP) of the European Medicines Agency (EMA) with an estimated incidence of 1.6/10,000 based on the published epidemiological data available on the main conditions associated with NK^4^.

Ocular surface injuries cause the corneal sensory nerves to react through ocular symptoms of pain, irritation and triggering protective reflexes such as blinking and tearing^3^. In fact, tears play a key role in maintaining healthy ocular surface providing growth factors and other nutrients after stimulation by the neuro-secretory reflex^6^. In general, therapeutic approaches for NK should target corneal innervations impairment in order to restore corneal integrity^3,4^. The administration of human recombinant nerve growth factor (*rhNGF*, cenegermin) eye drops, as a licensed-in-Europe specific medication for treatment of moderate (stage 2) and severe NK (stage 3), has added promising perspectives to the management of this clinical condition providing a valid alternative to the neurotization surgery in a number of cases^7^. In fact, *rhNGF* is able to replace nerve grow factor and promotes nerve regeneration, reduces epithelial cells apoptosis encouraging epithelium healing^1,8,9^ and, finally, results beneficial in recovering corneal damage by restoring corneal sensations^7,10,11^. In light of these growing therapeutic evidences the use of cenegermin (OXERVATE™) has been approved in both the European Union for stages 2 and 3 NK and in the United States for all stages of NK^7^. However, to date, few studies have been conducted to monitor response to treatment from a molecular point of view, thus more detailed knowledge about the mechanisms underlying the response to the treatment are necessary.

Within such a complex context, tear film could represent an elective source of diagnostic and prognostic biomarkers for the investigation of ocular and no-ocular neurodegenerative diseases^12–16^. The main advantage in the use of tears as a biological fluid lies in their relatively easy collection, particularly suitable for therapeutic monitoring applications and, nevertheless, their proximity to the Central Nervous System. The attention on the molecular study of this fluid is increasingly growing among researchers. At the same time, “omics” tear film investigation could offer a broad overview of its molecular alterations^13,14,16^. In the last decade, “omics” approaches are considered a promising tool both to reveal molecular pathways and to identify and quantify different molecules expressed in many pathophysiological contexts, and specifically proteomics allows the characterization of the expression, structure, functions, interactions and modifications of proteins at any stage^17^. Nowadays, proteome characterization is greatly accelerated thanks to the improvements in reverse phase chromatography coupled to Mass Spectrometry (MS), and thanks to the use of high-resolution MS analyzers, obtaining an extraordinary resolving power and fast duty cycles, applicable even for complex samples as biological fluids^18^. Furthermore, in the field of research on biological fluids, attention is paid to phenotyping and counting of circulating Extracellular Vesicles (EVs), considered a heterogeneous group of membranous structures derived from cells. In the past, EVs were only considered waste carriers, but in recent years they have emerged as functional signaling vehicles^19^.

In the present work, we proposed a label-free proteomics approach to investigate tear proteome in rare NK patients at baseline and in the follow-up period, specifically after 4 weeks of topical *rhNGF* treatment and at the end of the treatment, after 8 weeks, in order to highlight the molecular repercussions of pharmacological treatment and to find new molecular markers of response to the therapeutic. In vivo confocal microscopy analysis and phenotyping of tears EVs^20^ have been acquired, confirming proteomics data on the neuronal response induced by the drug.

## Results

### Tear proteomics during rhNGF treatment

**Figure 1:**
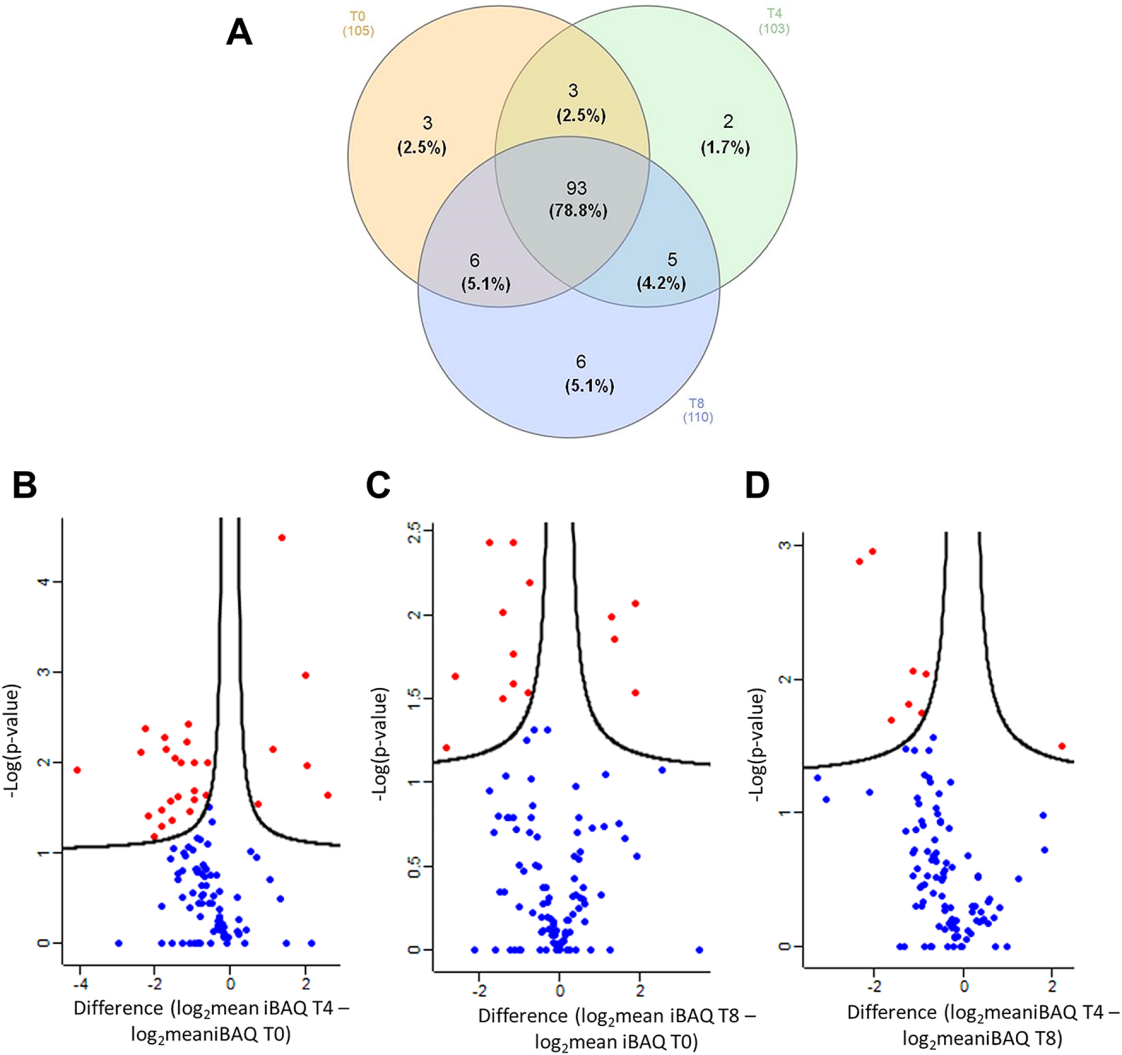
Tears proteins identification and statistic. (**A**) Venn diagram of quantified proteins in tears of NK patients at baseline (T0), after 4 (T4) and 8 weeks (T8) of treatment with *rhNGF*. Volcano Plot of proteins graphed by fold change (Difference) and − Log(P value) by the comparison of T4 vs T0 (**B**); T8 vs T0 (**C**); T4 vs T8 (**D**). Blue dots represent proteins that were not differentially expressed in the comparison carried out; red dots represent proteins that are significantly up-regulated (right dots in the Volcano plot) or down-regulated (left dots in the plot) in each investigated comparison.

Shotgun proteomics investigation was carried out to compare tear proteins expression in NK patients at baseline (T0), after 4 and 8 weeks of treatment with *rhNGF* (T4andT8, respectively). Venn diagram in Fig. 1A shows the summary of the proteomics results obtained in term of the number of common and unique proteins identified in the three conditions investigated. Details of such unique proteins for each condition are reported in the Table 1. In particular, Neutrophil elastase (*ELANE*), a secreted protein by neutrophils and macrophages during inflammation^21^, and Beta-actin-like protein 2 (*ACTBL2*) appear as unique proteins after 4 weeks of treatment. Moreover, at the end of treatment six unique proteins are identified: protein/nucleic acid deglycase DJ-1, Nucleobindin-2, WAP four-disulfide core domain protein 2, Alpha-1B-glycoprotein, Lactoperoxidase, Selenium-binding protein 1 (*PARK7, NUCB2, WFDC2, A1BG, LPO* and *SELENBP1*). Alongside these unique proteins, we compared the expression of common proteins at each investigated timepoint in respect to T0 point, as reported in the Volcano plots of Fig. 1B,C. In particular, Fig. 1B shows the ratio between the protein expression after 4 weeks of treatment with *rhNGF* in respect to naïve tears, while Fig. 1C shows the ratio between protein expression at the end of treatment compared to naïve tears protein, highlighting 28 and 15 significant differential proteins respectively. Figure 1D shows 8 significant differential proteins in the comparison of the T4 vs T8 timepoint. In particular, red dots represent proteins that are significantly up-regulated (right) or down-regulated (left) in T4 vs T0 (Panel B), in T8 vs T0 (Panel C)and in T4 vs T8 (Panel D).

**Table 1:** Unique proteins identified in the three different clinical conditions: at baseline (T0), after 4 (T4) and 8 weeks (T8) of treatment with *rhNGF*.

Gene name	Description	T0	T4	T8
AKR1A1	Alcohol dehydrogenase	+		
CAP1	Adenylylcyclase-associatedprotein 1	+		
YWHAB	14–3–3 protein beta/alpha	+		
ELANE	Neutrophilelastase		+	
ACTBL2	Beta-actin-like protein 2		+	
PARK7	Protein/nucleic acid deglycase DJ-1			+
NUCB2	Nucleobindin-2			+
WFDC2	WAP four-disulfide core domain protein 2			+
A1BG	Alpha-1B-glycoprotein			+
LPO	Lactoperoxidase			+
SELENBP1	Selenium-bindingprotein 1			+

### Tear proteins expression after 4 weeks of rhNGF treatment delineates an inflammation and neuro-regeneration state

**Figure 2:**
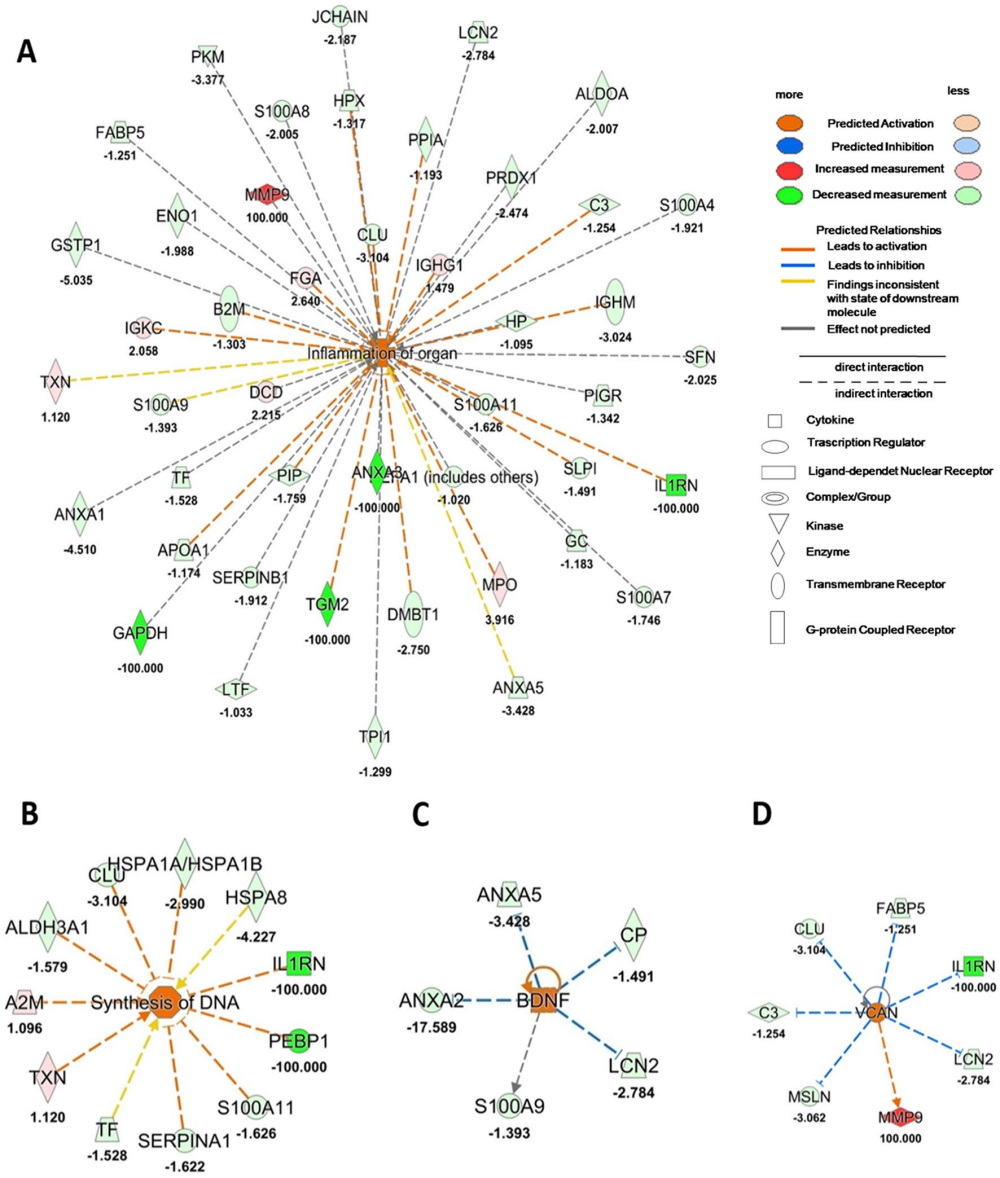
Tear functional proteomics results after 4 weeks of treatment. (**A**, **B**) Downstream analysis comparing proteomics dataset at T4 Vs T0 condition. The most significant downstream were: *Inflammation of organ* (**A**) and *DNA synthesis* (**B**). (**C**, **D**) reported the most significant upstream comparing proteomics dataset at T4 Vs T0 condition: *Brain-derived neurotrophic factor (BDNF)* (**C**) and *Versican Core Protein (VCAN)* (**D**). Orange or blue shapes represent predicted activation (z-scores ≥ 2.0) or predicted inhibition (z-scores ≤ 2.0), respectively. The color intensity is directly proportional to the significance of the predicted activation or inhibition. The predicted relationship between genes may lead to direct activation (orange solid lines) or direct inhibition (blue solid lines). Instead, red and green shapes represent increased or decreased measurements of identified proteins, respectively (whose fold change value is reported in the figure). Colour key and symbols are reported in the figure.

Quantified proteins ratio in tears between naïve NK patients and after 4 weeks of *rhNGF* treatment was used as input for functional analysis. Figure 2A,B reports the results of downstream analysis, showing as tear protein expression after 4 weeks of treatment explains both a deep *Inflammation of organ* (p-value = 2.43 × 10^–19^, z-score = 2.7) (Fig. 2A), according with clinical symptoms, and allows the prediction of new *DNA Synthesis* (p-value = 5.97 × 10^–5^, z-score = 2.08), as reported in the network depicted in Fig. 2B. Furthermore, upstream analysis of the same datasetreported in Fig. 2C,D, reveals*Brain-derived neurotrophic factor (BDNF)* and *VersicanCoreProtein (VCAN)* pathways as significantly activated by showing a z-score of 2.0 and 2.63, respectively.

### Tear proteome at 8 weeks of rhNGF treatment proves an activation ofInterleukin-6 receptor during therapy

**Figure 3:**
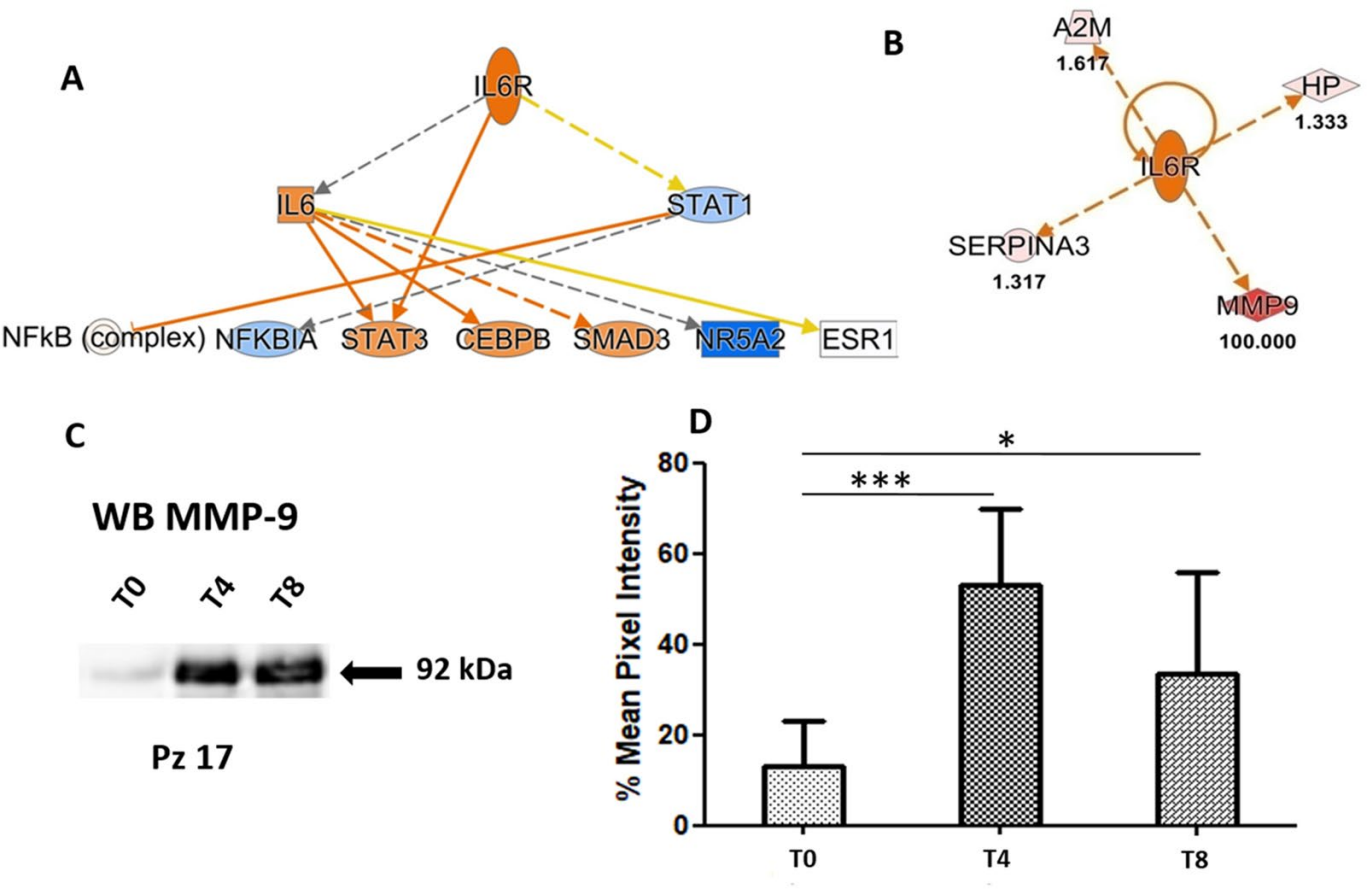
Tear functional proteomics results after 8 weeks of treatment. (**A**) *Interleukin-6 receptor (IL6R)* resulted the most significant activated Upstream by comparing proteomics dataset at T0 and T8 timepoint of treatment with *rhNGF*. (**B**) Identified proteins related to the activation of *IL6R*.Colour key and symbols are reported in the Fig. 2. (**C**) *Matrix metalloproteinase-9 (MMP-9)* expression in one representative NK patient at T0, T4 and T8 timepoint during therapy. (**D**) Histograms reporting the mean of six patients of OD pixel intensity in percent of the *MMP-9* band (92 kDa). *MMP-9* expression in each patient at T0, T4 and T8 is reported in the WBs of Supplementary Fig. S1. Multiple exposure time points, as well as the full-length blots are shown in Figs. S2 and S3. Error bars show the standard error of the mean from six NK patients at the different times of treatment. * means p-value < 0.05, *** means p-value < 0.001 at the Student’s t-test.

The same experimental scheme, described in the previous paragraph, was carried out to functionally describe the effects of *rhNGF* on the lacrimal proteome in NK disease after 8 weeks of treatment. Thus, quantified proteins ratio in tears of naïve NK patients and after 8 weeks of treatment was used as input for functional analysis. In particular, upstream analysis allowed to predict a strong activation of *Interleukin-6 receptor (IL6R)* in treated patients after 8 weeks of treatment (Fig. 3A)*.* The predicted significant activation of *IL6R*, obtained by “in silico” upstream investigation, is mainly determined by a very high expression of *Matrix metalloproteinase-9 (MMP-9)* found in tear during treatment. Actually, as reported in Fig. 3B, *MMP-9* is one of the most abundant identified proteins in our dataset, that is related to an activation of IL6*.*The up-regulation of *MMP-9* during treatment has been validated by Western Blot (WB) as reported in Fig. 3C where a representative sample is shown. WB of *MMP-9* expression in patients at baseline and at different times of treatment is displayed in Supplementary Fig. S1. Multiple exposure time points, as well as the full-length blots are shown in Figs. S2 and S3. While Fig. 3D shows the mean of the percentage of variation in *MMP-9* expression at the three different times analysed.

### Inflammation response predicted by tear proteomics is mediated by specific sub-type of extracellular vesicles

**Figure 4:**
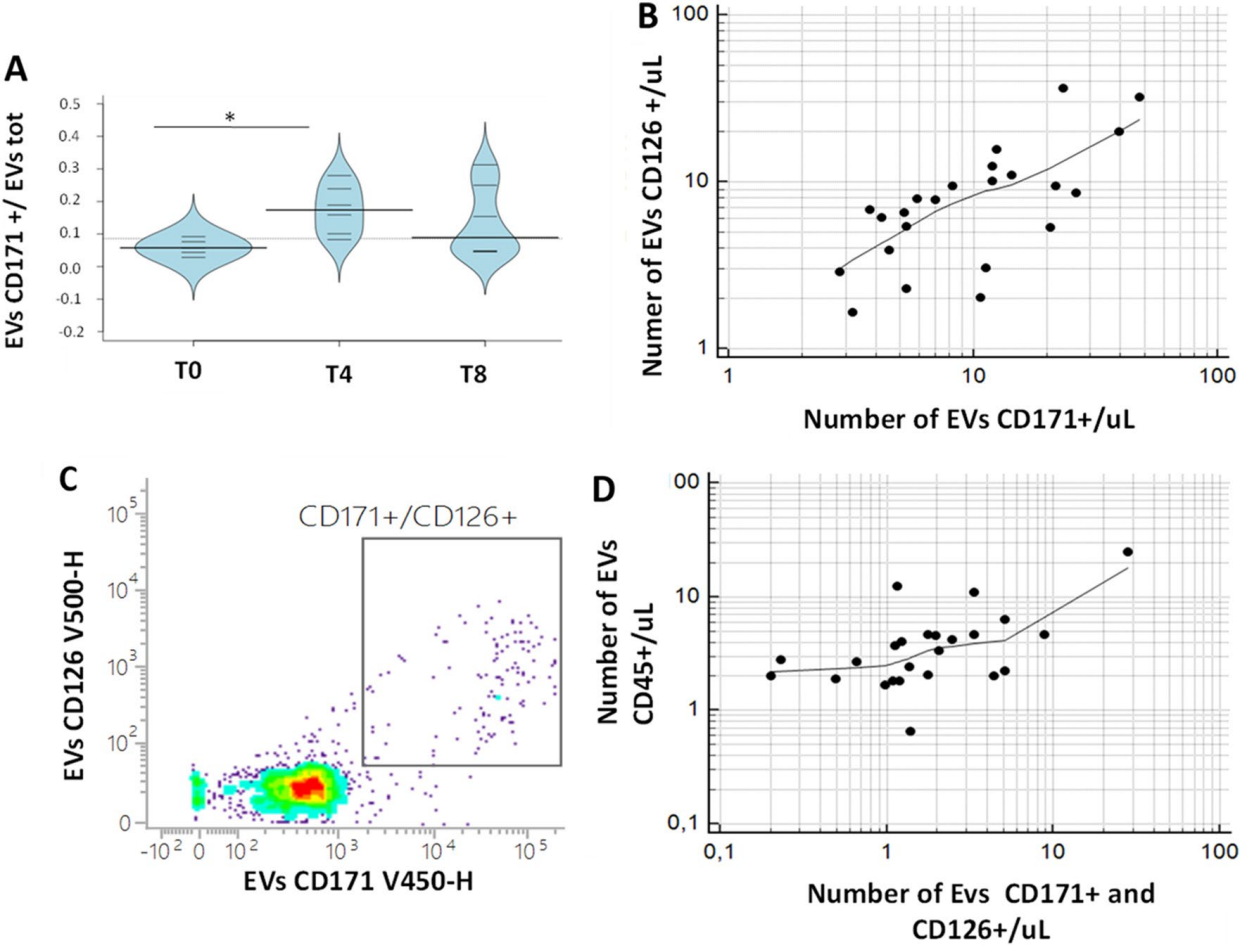
EVs characterization in tears during *rhNGF* treatment. (**A**) Bean plots reporting the distribution of relative numbers of EVs CD171 + (ratio between EVs CD171+ and total EVs) in the comparison between tear samples collected during *rhNGF* treatment. The polygon shape (in light blue) represents the density trace of each variable, and inside to that, a scatter plot shows all individual measurements for each single NK patient (black lines). * means p-value < 0.05, at the Student’s t-test and/or Mann Whitney U-test. (**B**) Rank correlation (Spearman’s coefficient of Rank correlation (rho) = 0.71, and p = 0.0001) between the number of EVs CD171+ and EVs 126+ counted and gated in tears samples collected during *rhNGF* treatment. (**C**) The CD171/CD126 dot-plot shows the gate identifying the CD171+/CD126+ population of EVs. (**D**) Rank correlation (Spearman’s coefficient of Rank correlation (rho) = 0.54, and p = 0.0067) between double positive EVsCD171+/CD126+ with EVs CD45+ in tears samples collected during *rhNGF* treatment.

To deeply investigate the biological machinery involved in neuronal and inflammatory response during *rhNGF* treatment, numbers and subtypes of lacrimal EVs were analysed through a patented flow-cytometry method previously described^19,20,22^. In particular, leukocyte-derived EVs (CD45+) and neuronal-derived EVs (CD171+) were evaluated in tears samples collected at T0, T4 and T8 of *rhNGF* treatment. Moreover, EVs exhibiting Interleukin-6 receptor (CD126+) were gated and counted in naïve tears samples, as well as during treatment. As reported in Fig. 4A, a significantly increasing of the relative numbers of neuronal EVs CD171+ were observed in the comparison between tears of patients after 4 weeks of treatment with *rhNGF* in respect to naïve tears (p-value < 0.05). The same trend for EVs CD126+ was observed, albeit not statistically significant (data not shown). Furthermore, a significant correlation between the count of neuronal EVs CD171+ and EVs exhibiting Interleukin-6 receptor CD126+ was observed in tears of patients collected during *rhNGF* treatment, as reported in Fig. 4B (Spearman’s coefficient of rank correlation rho = 0.71 and p-value = 0.0001). To verify whether neurotrophic and inflammatory stimuli are transported within the same biological recipient, subtypes of lacrimal EVs expressing both neuronal phenotype (CD171+) and Interleukin-6 receptor (CD126+) were gated and counted. Our data demonstrated the effective presence of EVs which carry both neuronal marker and IL6 receptor, as reported in Fig. 4C. Moreover, these double positive EVs highlighted a very strong correlation with CD45+ EVs, as depicted in Fig. 4D, with a Spearman’s coefficient of rank correlation (rho) = 0.54 and p-value = 0.007. In addition, as reported in Supplementary Fig. S4, the correlation was stronger in treated samples than the naïve ones, indicating the key role of *rhNGF* treatment in double positive EVs release and function.

### In vivo confocal microscopy analysis corroborates proteomics results

**Figure 5:**
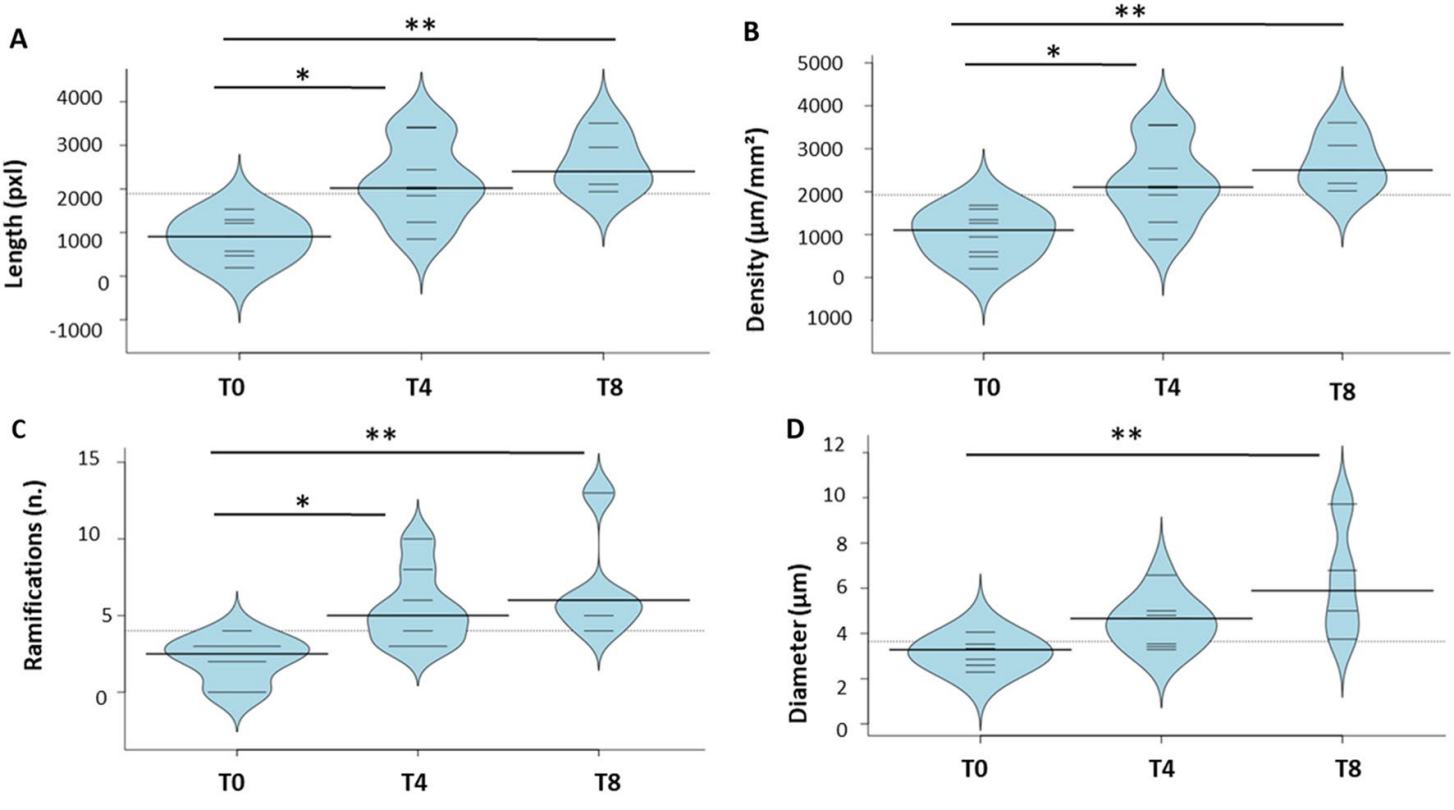
Clinical confirmation of biochemical results. Distribution of clinical data: fiber length (**A**), nerve density (**B**), number of nerve ramifications (**C**) and diameter (**D**) of nerve fibers. Data are visualized as bean plots where the polygon shape (in black line and light blue filling) represents the density trace computed using a log- transformation of each variable, instead a scatter plot shows each single NK patient used for the distribution (in black line).* means p < 0.05, and ** means p < 0.01 obtained by the Kruskal–Wallis test and Dunns’ post hoc test.

**Figure 6:**
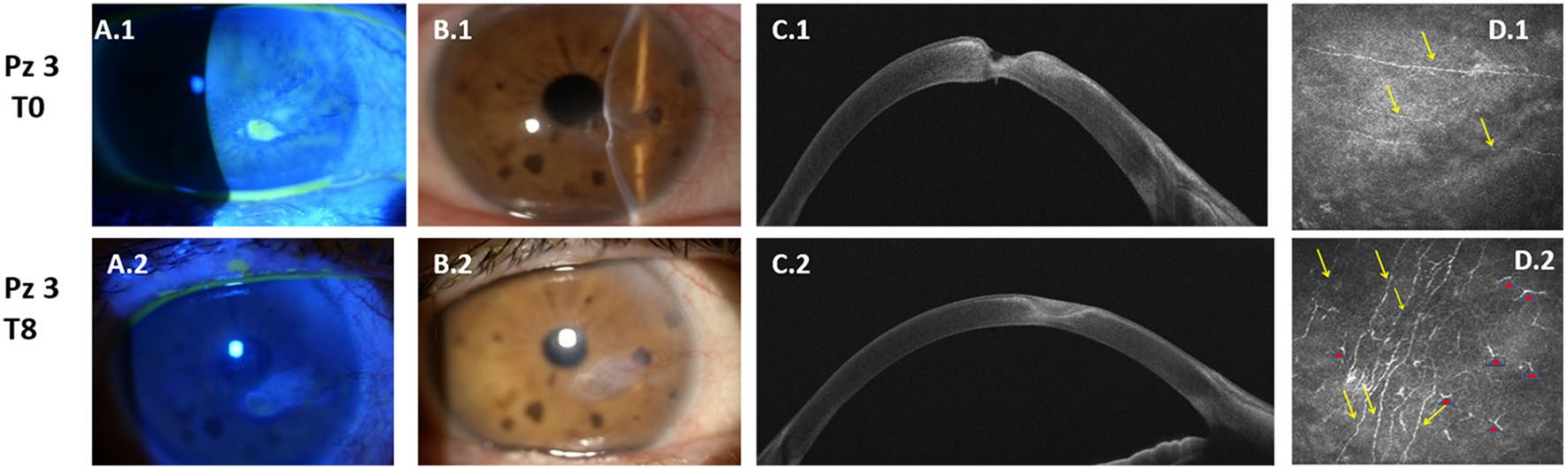
Representative case of severe NK before and after treatment. Patient with an evident corneal ulcer, evaluated by slit lamp examination and fluorescein staining (**A1**–**B1**). Anterior segment OCT shows an important stromal thinning (**C1**) and in vivo confocal microscopy analysis (IVCM) (**D1**) evidences a reduced sub-basal nerve fiber density. After 8 weeks of treatment with topical *rhNGF*, the resolution of the epithelial defect was evident at slit lamp examination and fluoresce in staining (**A2**–**B2**), the healing of stromal ulceration was evident at Anterior segment OCT (**C2**) and IVCM shows an evident increase of subepithelial nerve fiber density together with Dendritic Cells (DCs) at the level of basal epithelium (**D2**). Yellow arrows show nerve fibers, while red triangles show DCs.

Clinical in vivo evaluations on the same patients and at the same time points confirm the biochemical results obtained, as reported in Fig. 5. The length (Panel A), density (Panel B) and number of ramifications (Panel C) of nerve fibers increase during treatment (T4) (p-value < 0.05) and, more evidently (p-value < 0.01) at the end of the therapeutic course (T8). Remarkably, the nerve fibers diameter (Panel D) increases significantly at the end of the NGF treatment (p-value < 0.01). A representative case of the healing of a case of severe NK obtained with topical *rhNGF* treatment is reported in Fig. 6. The epithelial restoration in Panels B2–C2 and stromal healing in Panel D2 were associated with an increase of nerve fiber density and dendritic cells visualized at the level of the basal epithelium by means of IVCM. These results are correlated to the increased inflammation pathways predicted by proteomics data (Fig. 3) and most likely mediated by EVs showed in Fig. 4. Actually, the increased number of nervefibers in respect to naïve ones as shown in Fig. 6D1 is in accordance to the high number of neuronal CD171+ EVs.

## Discussion

NK patients present good healing in term of length, density and number of ramifications of nerve fibers after *rhNGF* treatment. In support of these evidences, already described in a different casuistry^7^ and confirmed also in our clinical group, a functional proteomics study in lacrimal fluid during therapy was carried out in a precious group of patients. Due to the low incidence of such orphan disease, the number of patients was limited and almost the half of them stopped early the therapy, someone for significant inflammatory side effects, others for different reasons. From 15 enrolled subjects, eight of them completed the study. Proteomics results on pooled tear samples, demonstrated how the typical tear proteins, in each timepoint analysed, reflect the evaluated clinical state. Actuality, *CAP1* and *YWHAB*, only quantified at the baseline T0, are involved in protein binding and membrane organization. On the other hand, *ELANE* and *ACTBL2* are specific proteins quantified only after 4 weeks of treatment T4. In particular, *ELANE,* a Neutrophil elastase*,* plays a regulation role in inflammatory response^21,23^. Actually, our functional and in vivo data demonstrated a condition of severe inflammation in patients with NK during the treatment with *rhNGF* and *ELANE* over-expression could be associated to a specific triggered inflammation mechanism in response to the treatment. On the other hand, *ACTBL2* is strongly related to cell motility and growth events highlighting that *rhNGF* treatment allows an increase in thenumber of nerve fibers and their ramifications.

These evidences are corroborated by upstream regulators analysis based on our proteomics dataset showing as, after 4 weeks of treatment, the proteins expression in tears is driven by activation of *BDNF* and *VCAN* (activated upstream). The numerous effects of *BDNF* on the Nervous System include axonal regeneration, plasticity, and re-myelination^24^ and could be correlated with the morphological improvements described in these patients. Moreover, Namiki et al. indicate that invasion and proliferation of Schwann cells and formation of peripheral myelin were more prominent at the injury site in the *BDNF*-treated animals than in the other groups^25^. Furthermore, *VCAN* gene was demonstrated to be conserved between human developing retina and human pluripotent stem cell-derived retinal organoids^26^, and this could justify its activation during neurons remodelling in *rhNGF* treatment. The importance of such gene in eye health is proved by the evidence that Wagner syndrome, a rare inherited vitreoretinopathy, is caused by mutations in the canonical consensus sites of *VCAN*. Despite the mobilization of such regeneration factors, treatment with *rhNGF* reveals also high in situ inflammation, well known clinically, but not described so far from a molecular point of view, as reported by the downstream pathways highlighted.

Regarding the modulation of the tear proteomeat the end of treatment, the six unique proteins quantified at T8 are mainly related to neuroprotection, immunity response and inflammation. In particular, *PARK7* is involved in neuroprotective mechanisms and has an important role in antioxidative stress to prevent cell death^27^, and *SELENBP1* may also be involved in neurite growth and remodelling^28^. In addition, we demonstrated how after 8 weeks of *rhNGF* treatment tear proteins are working to reduce the inflammatory condition, as demonstrated by the presence of *NUCB2*, which is involved in the suppression of inflammation after traumatic brain injury^29^. Moreover, *WFDC2* and *LPO* play an important role in innate immune defence.

Interestingly, after 8 weeks of treatment, upstream analysis allowed to predict a strong activation of *IL6R*, suggesting a major susceptibility to the inflammatory stimulus even if the levels of circulating IL6 resulted unchanged (data not shown). The activation of *IL6R* was confirmed by an up-regulation of *MMP-9* that is triggered by innate inflammatory mediators as extensively proven^30^, so that the inhibition of *MMP-9* as therapeutic target for treatment of Dry Eye Syndrome, was recently suggested^31^.

We analysed and counted neuronal derived EVs in tears following a patented flow-cytometry method already described in our previously work^19^. Cytofluorimetric results on neuronal regeneration and inflammatory response during *rhNGF* treatment have confirmed the functional proteomics data obtained.

In particular, we found that the number of neuronal EVs (CD171+) increases during treatment and were significantly correlated with the increase of EVs expressing IL6R (CD126+). Furthermore, the number of neuronal-derived EVs expressing IL6R (double positive for CD171 and CD126) increases proportionally to leukocyte-derived CD45+ EVs, suggesting that, specific EVs mediating a balanced response between inflammation and neuronal regeneration are released during treatment.

In summary, the up-regulation of *IL6R*at the end of *rhNGF* treatment is probably powered by released EVs by growing neuronswhich express such receptor on their surface. Actually, it was demonstrated as neuronal CD171+ EVs are associated to a marked increase in cell proliferation, as determined by DNA cell cycle analysis and cell counting on Glioma and as chemical inhibitors against *fibroblast growth factor receptor (FGFR)* decreased CD171-mediated motility and proliferation to varying degrees^32^.

Proteomics and cytofluorimetric results were in vivo validated in term of length, density and number of ramifications of nerve fibers increased during treatment, whereas the diameter of nerve fibers increased significantly at the end of the *rhNGF* treatment. The epithelial restoration and stromal healing during treatment are related both to an increase of inflammation pathways mediated by EVs andan increase of nerve fiber density.

In conclusion, our data showed, for the first time, evidence on molecular pathways involved in the response to therapy of NK patients treated with *rhNGF* eye drops. The main evidence regards a therapeutic effect during the treatment combining neuro-regeneration and inflammation mechanisms that in some cases determined a severe inflammatory clinical condition (causing, in some subjects, the voluntary interruption of the treatment) that trigger a neuro-regeneration resolution. These data will contribute to better understand the biological pathways underlying the therapeutic effect of *rhNGF* in the treatment of NK that could be useful in the assessment of drug response and to predict/ameliorate the clinical outcome.

## Methods

### Patient enrollment and clinical evaluation during treatment

In this prospective observational case series 15 patients (7 males and 8 females) with documented moderate or severe NK were enrolled. Only 8 of 15 finished therapy by undergoing follow-up checks until the eighth week of starting treatment. Informed consent was obtained from all patients who were selected for our study. The study design was made following the protocol approved on 29 December 2020 by the Ethic committee of “G. d’Annunzio” University, in accordancewith the Declaration of Helsinki (World Medical Association, 1997). All patients were treated with cenegermin 20 µg/mL (OXERVATE™, Dompè Farmaceutici Spa, Milan, Italy) in the affected eye 1 drop every 2 h 6 times per day for 8 weeks. Contact lens use was discontinued. Slit lamp examination, Schirmer 1 test, and In Vivo Confocal Microscopy (ICVM) were performed at baseline and after 4 and 8 weeks of follow-up. IVCM was performed by using a confocal microscope diode-laser 670 nm (HRT II Rostock Cornea Module; Heidelberg Engineering, Heidelberg, Germany). IVCM scans were focused on the central cornea. At least five frames at the level of the epithelium and basal lamina were acquired. Corneal sub basal nerve density was traced using NeuronJ, a free semi-automated analysis plug-in program of ImageJ (National Institutes of Health, Bethesda, Maryland, USA). The density of the nerve fibers was calculated in µm/mm^2^. The number of nerve branches and the nerve diameter was manually quantified in each frame and reported as µm and number/frame. All the IVCM nerve fibers parameters were evaluated as an average of five different scans acquired in central cornea. At the level of basal epithelial layers, basal lamina, or sub-basal nerve plexus layer, the presence of DCs was investigated. DCs were identified as bright cellular images with a branching dendritic morphology. DCs density was calculated, using the analysis software provided by the microscope instrument, by averaging numbers of cells from five images acquired in central cornea, counted manually within a region of interest (ROI). DCs densities are given a cells/mm^2^.

### Tear samples collection and extraction for proteomics study

Tears samples were collected at the Ophthalmology Clinic of the University “G. d’Annunzio” of Chieti-Pescara (Italy) on Schirmer strips purchased from EasyOpht (Busto Arsizio, VA, Italy), as we previously described^13,14^. Tears were collected on graduated paper strips pulling the lower eyelid gently downward for 5 min. Subsequently, they were placed in 2.0 mL Eppendorf tube and stored at − 80 °C. Tear Schirmer’s strip samples were extracted as described in our previous work^16^ and used for proteomics investigation or for subsequent Flow Cytometry EVs phenotyping or/and WB analyses. Proteomics analyses were executed on whole lacrimal fluid from 8 subjects suffering from NK at baseline (T0) and after 4 (T4) and 8 weeks (T8) of treatment with *rhNGF*, that is cenegermin 20 mg/mL. Tear proteins were first extracted, quantified by Bradford assay (Bio-Rad, Hercules, CA, USA), using Bovine Serum Albumin (BSA, Sigma-Aldrich, St. Louis, MI, USA) standards for the calibration curve, and then grouped, in order to obtain the samples for the three treatment monitoring steps chosen for the proteomics study (T0; T4 and T8). 30 µg of proteins underwent to a tryptic digestion carried out overnight at 37 °C using trypsin (Merck KGaA, Darmstadt, Germany) by performing a filter-aided sample preparation (FASP) protocol.

### Proteomics analysis and data processing

Tryptic peptides from each sample were analyzed in triplicate by LC–MS/MS using a Proxeon EASY-nLCII (Thermo Fisher Scientific, Milan, Italy) chromatographic system coupled to a Maxis HD UHR-TOF (Bruker Daltonics GmbH, Bremen, Germany) mass spectrometer, as already described in our previous works^16,19^.

LC–MS/MS data were processed simultaneously using a free computational proteomics platform, MaxQuant version 1.6.6.0 (Max-Planck Institute for Biochemistry, Martinsried, Germany). Peak lists, generated in MaxQuant, were searched using Andromeda peptide search engine against the UniProt database (released 2018_04, taxonomy *Homo sapiens*, 20,874 entries) supplemented with frequently observed contaminants and containing forward and reverse sequences. Multiplicity was set to one because a label-free quantification was performed. Trypsin digestion was specified as digestion mode. Non-specific cleavage to both ends of the peptides was allowed with maximum of two missed cleavages. Carbamidomethylation of cysteines (C) was defined as fixed modification and used in protein quantification, while oxidation of methionines (M) and deamidation of asparagines and glutamines (NQ) were set as variable modifications. Minimum peptide length of 7 amino acids was set and the search space was limited to a maximum peptide mass of 4600 Da. MaxQuant uses individual mass tolerances for each peptide; the initial maximum precursor mass tolerances were set by default to 0.07 Da in the first search and 0.006 Da in the main search, and the fragment mass tolerance was set to 0.05 Da. Match-between-runs (MBR) algorithm was used to transfer the peptide identifications from one LC–MS/MS run to all others using its default settings (match window of 0.7 min and alignment time of 20 min). False discovery rate (FDR) at protein level was set at 2%, on the contrary at peptide level was set at 1%. Protein identification was performed with at least one unique peptide. Protein abundance of each sample was measured as intensity-based absolute quantification (iBAQ) and used for functional enrichment analysis.

The mass spectrometry proteomics data have been deposited to the ProteomeXchange Consortium via the PRIDE partner^33^ repository with the dataset identifier PXD025408.

### Bioinformatics and functional analysis

Bioinformatics analyses were performed using Perseus software, version 1.6.10.50, (Max-Planck Institute for Biochemistry, Martinsried, Germany) uploading the identified protein groups generated by MaxQuant. First of all, data were log_2_ transformed in order to facilitate the calculation of the protein expression. Site only, reverse and contaminant peptides were removed from the dataset. Then, the missing and invalid values were removed. The minimum number of valid values accepted was set at 2 in at least one clinical group. In this way we have evaluated not only the different protein expression, but also the presence and absence of proteins between two different clinical conditions.

Variability between different clinical groups is reported as Pearson correlation (R^2^) as log_2_ values in a density plot (Supplementary Fig. S5). The Volcano plot function was used to identify the differentially regulated proteins by performing a T-test (p-value < 0.05) with a false discovery rate (FDR) of 0.1 and S_0_of 0.1 for T4/T0 comparison and a FDR of 0.2 and a S_0_of 0.1 for T8/T0.

At last, obtained protein datasets were further uploaded for “Core Analysis” through Ingenuity Pathway Analysis tool (IPA, Quiagen, Hilden, Germany) to map the modulated proteins for their functional annotation, such as canonical pathway analysis, network discovery, upstream regulator analysis and downstream effects networks. Protein ratio was uploaded for each defined comparison, in which we considered molecules and/or relationships in all species and a confidence setting as high predicted or experimental observed (excluding medium predicted). IPA is able to identify relationships and pathways relevant to the uploaded dataset. In particular, it provides the principal diseases and function categories resulting from some of the modulated proteins of the uploaded dataset. Instead, upstream regulator analysis is based on prior knowledge of expected effects and relationships between transcriptional regulators and their target genes from published literature citations stored in the IPA system^34^. The predicted activation or inhibition of each transcriptional regulator or downstream is inferred by the z-score generated by IPA system (z-scores ≥ 2.0 indicate that a molecule is activated, whereas z-scores ≤ − 2.0 indicate the inhibition of target molecules). Instead, the p-value is a measurement of the statistical overlap between the protein dataset and the genes or function categories, and the significance is attributed to p-value < 0.05.

### Western Blotting

After tears extraction and protein quantification by Bradford assay, tear proteins were separated by 10% SDS-PAGE, transferred to a nitrocellulose membrane, and then blocked for 1 h at room temperature using blocking buffer (5% not-fat dry milk in PBS-Tween20 0.01%). Thus, membranes were incubated with primary anti-*MMP-9* (EP1255Y) antibody used 1:1000 (Novus Biologicals, Centennial, Colorado, USA) overnight at 4 °C. Next, membranes were washed with PBS-Tween20 0.01% for three times and incubated with rabbit horseradish peroxidase-conjugated secondary antibody, diluted 1:20,000, for 1 h at room temperature. Visualization was achieved using the enhanced chemiluminescence method (SuperSignal West Dura Extended Duration Substrate, Thermo Fisher Scientific, USA). WB of *MMP-9* expression at baseline and at different times of treatment is shown in Supplementary Fig. S1. The optical density of WB bands was assessed through Image J software (National Institute of Mental Health, USA) by calculating % Mean of Pixel Intensity.

### Flow cytometry detection and subtyping of extracellular vesicles

Tear samples were analysed as previously reported^16,19,20,35^. Briefly, 100 μL of tears were stained using a reagent mix, as detailed in Table S1. Samples were then incubated for 45 min, at room temperature, in the dark. After adding 200 μL of PBS 1X to each tube, 1 × 10^6^ events/sample were acquired by a FACSVerse flow cytometer (BD Biosciences). The trigger threshold was set on the allophycocyanin (APC) channel, which is the channel in which the LCD (a pan EV marker) emits (threshold placed at 200/262,144)^20,36^. For all used parameters the height (H) signals and the bi-exponential/logarithmic modes were selected. Current guidelines for flow cytometry analyses and for EV studies were taken into account^37,38^. The Cytometer Setup & Tracking Module (BD Biosciences) was used for the daily quality check. Compensation was assessed using CompBeads (BD Biosciences) and single stained fluorescent samples. Data were analysed using FACSuite v 1.0.6.5230 (BD Biosciences) and FlowJo X v 10.0.7 (TreeStar, Ashland, OR, USA) software. EVs concentrations were obtained by the volumetric count function. With the used dilution no swarm effects occurred.

### Statistical analysis

Kruskal–Wallis test with Dunn’s Multiple Comparison post-test was performed for comparisons between the three different clinical groups, using GraphPad Prism (GraphPad software, Inc. USA). Rank correlation between variables were performed using MedCalc 19.4.1 (MedCalc software Ltd), by using a Spearman non-parametric correlation test. The values of p < 0.05 were considered significant. The 95% of confidence interval was assumed for each test. BoxPlot R was used to perform bean plots for clinical data distribution visualization.

## Supplementary Information

Supplementary Information.

## References

[1] Dua HS (2018). Neurotrophic keratopathy. Progress Retinal Eye Res..

[2] Mastropasqua L, Nubile M, Lanzini M, Calienno R, Dua HS (2019). In vivo microscopic and optical coherence tomography classification of neurotrophic keratopathy. J. Cell. Physiol..

[3] Mastropasqua L, Massaro-Giordano G, Nubile M, Sacchetti M (2017). Understanding the pathogenesis of neurotrophic keratitis: The role of corneal nerves. J. Cell. Physiol..

[4] Sacchetti M, Lambiase A (2014). Diagnosis and management of neurotrophic keratitis. Clin. Ophthalmol..

[5] Bonini S, Rama P, Olzi D, Lambiase A (2003). Neurotrophic keratitis. Eye.

[6] Meng ID, Kurose M (2013). The role of corneal afferent neurons in regulating tears under normal and dry eye conditions. Exp. Eye Res..

[7] Mastropasqua L (2020). In vivo evaluation of corneal nerves and epithelial healing after treatment with recombinant nerve growth factor for neurotrophic keratopathy. Am. J. Ophthalmol..

[8] Bonini S (2018). Phase I trial of recombinant human nerve growth factor for neurotrophic keratitis. Ophthalmology.

[9] Bonini S (2018). Phase II randomized, double-masked, vehicle-controlled trial of recombinant human nerve growth factor for neurotrophic keratitis. Ophthalmology.

[10] Chang EJ (2008). The role of nerve growth factor in hyperosmolar stress induced apoptosis. J. Cell. Physiol..

[11] Di Zazzo A, Varacalli G, Mori T, Coassin M (2020). Long-term restoration of corneal sensitivity in neurotrophic keratopathy after rhNGF treatment. Eur. J. Ophthalmol..

[12] Agnifili L (2015). Molecular biomarkers in primary open-angle glaucoma: from noninvasive to invasive. Progress Brain Res..

[13] Cicalini I (2019). Integrated lipidomics and metabolomics analysis of tears in multiple sclerosis: An insight into diagnostic potential of lacrimal fluid. Int. J. Mol. Sci..

[14] Pieragostino D (2017). Tear film steroid profiling in dry eye disease by liquid chromatography tandem mass spectrometry. Int. J. Mol. Sci..

[15] Pieragostino D (2015). Unraveling the molecular repertoire of tears as a source of biomarkers: Beyond ocular diseases. Proteom. Clin. Appl..

[16] Rossi C (2019). Multi-omics approach for studying tears in treatment-naive glaucoma patients. Int. J. Mol. Sci..

[17] Aslam B, Basit M, Nisar MA, Khurshid M, Rasool MH (2017). Proteomics: Technologies and their applications. J. Chromatogr. Sci..

[18] Cufaro MC (2019). Extracellular vesicles and their potential use in monitoring cancer progression and therapy: The contribution of proteomics. J. Oncol..

[19] Pieragostino D (2019). Proteomics characterization of extracellular vesicles sorted by flow cytometry reveals a disease-specific molecular cross-talk from cerebrospinal fluid and tears in multiple sclerosis. J. Proteom..

[20] Marchisio M (2020). Flow cytometry analysis of circulating extracellular vesicle subtypes from fresh peripheral blood samples. Int. J. Mol. Sci..

[21] Belaaouaj A, Kim KS, Shapiro SD (2000). Degradation of outer membrane protein A in *Escherichia coli* killing by neutrophil elastase. Science.

[22] Brocco D (2019). Circulating cancer stem cell-derived extracellular vesicles as a novel biomarker for clinical outcome evaluation. J. Oncol..

[23] Tralau T, Meyer-Hoffert U, Schroder JM, Wiedow O (2004). Human leukocyte elastase and cathepsin G are specific inhibitors of C5a-dependent neutrophil enzyme release and chemotaxis. Exp. Dermatol..

[24] Weishaupt N, Blesch A, Fouad K (2012). BDNF: The career of a multifaceted neurotrophin in spinal cord injury. Exp. Neurol..

[25] Namiki J, Kojima A, Tator CH (2000). Effect of brain-derived neurotrophic factor, nerve growth factor, and neurotrophin-3 on functional recovery and regeneration after spinal cord injury in adult rats. J. Neurotrauma..

[26] Felemban M (2018). Extracellular matrix component expression in human pluripotent stem cell-derived retinal organoids recapitulates retinogenesis in vivo and reveals an important role for IMPG1 and CD44 in the development of photoreceptors and interphotoreceptor matrix. Acta Biomater..

[27] Taira T (2004). DJ-1 has a role in antioxidative stress to prevent cell death. EMBO Rep..

[28] Miyaguchi K (2004). Localization of selenium-binding protein at the tips of rapidly extending protrusions. Histochem. Cell Biol..

[29] Shimizu M (2020). Detection of NUCB2/nesfatin-1 in cerebrospinal fluid of multiple sclerosis patients. Aging.

[30] Pflugfelder SC, de Paiva CS (2017). The pathophysiology of dry eye disease: What we know and future directions for research. Ophthalmology.

[31] Shoari A, Kanavi MR, Rasaee MJ (2021). Inhibition of matrix metalloproteinase-9 for the treatment of dry eye syndrome; a review study. Exp. Eye Res..

[32] Pace KR, Dutt R, Galileo DS (2019). Exosomal L1CAM stimulates glioblastoma cell motility, proliferation, and invasiveness. Int. J. Mol. Sci..

[33] Perez-Riverol Y (2019). The PRIDE database and related tools and resources in 2019: Improving support for quantification data. Nucleic Acids Res..

[34] Kramer A, Green J, Pollard J, Tugendreich S (2014). Causal analysis approaches in Ingenuity pathway analysis. Bioinformatics.

[35] Pieragostino D (2018). Enhanced release of acid sphingomyelinase-enriched exosomes generates a lipidomics signature in CSF of Multiple Sclerosis patients. Sci. Rep..

[36] Simeone P (2020). Diameters and fluorescence calibration for extracellular vesicle analyses by flow cytometry. Int. J. Mol. Sci..

[37] Cossarizza A (2019). Guidelines for the use of flow cytometry and cell sorting in immunological studies (second edition). Eur. J. Immunol..

[38] Thery C (2018). Minimal information for studies of extracellular vesicles 2018 (MISEV2018): A position statement of the International Society for Extracellular Vesicles and update of the MISEV2014 guidelines. J. Extracell. Vesicles..

