# Supplementary material for: Tear proteomics reveals the molecular basis of the efficacy of human recombinant nerve growth factor treatment for Neurotrophic Keratopathy
Source: Sci Rep. 2022 Jan 24;12:1229. doi: 10.1038/s41598-022-05229-4 (PMC8786855; doi:10.1038/s41598-022-05229-4)

SUPPLEMENTARY MATERIALS OF:

Tear proteomics reveals the molecular basis of the efficacy of human recombinant nerve growth factor treatment for Neurotrophic Keratopathy

Damiana Pieragostino[1,2,*], Manuela Lanzini[3,4], Ilaria Cicalini[1,3], Maria Concetta Cufaro[1,5], Verena Damiani[1,2], Leonardo Mastropasqua[3,4], Vincenzo De Laurenzi[1,2], Mario Nubile[3,4],Paola Lanuti [1,3], Giuseppina Bologna[1,3], Luca Agnifili[3,4] and Piero Del Boccio[1,5]

[1]Center for Advanced Studies and Technology (CAST), University "G. d'Annunzio" of Chieti-Pescara, Italy.

[2]Department of Innovative Technologies in Medicine and Dentistry, University "G. d'Annunzio" of Chieti-Pescara, Italy.

[3]Department of Medicine and Aging Science, "G. d'Annunzio" University of Chieti-Pescara, Italy.

[4]Ophthalmology Clinic, National Centre of High Technology (CNAT) in Ophthalmology, University of "G d'Annunzio", Chieti-Pescara, Italy.

[5]Department of Pharmacy, University "G. d'Annunzio" of Chieti-Pescara, Chieti, Italy.

*Correspondence to:

Dr. Damiana Pieragostino

University "G. d'Annunzio" of Chieti-Pescara

Department of Innovative Technologies in Medicine and Dentistry

Via dei Vestini n. 31 66100 CHIETI

Tel. +39-0871-541593

e-mail: damiana.pieragostino@unich.it

Keywords: Proteomics; Tear; Neurotrophic Keratopathy; NGF; Extracellular Vesicles.

Table S1: List of flow cytometry specificities and reagents.

Detection	Fluorochrome	Vendor	Ab Clone	Catalog	Amount per Test
Phalloidin	FITC	BD Biosciences	-	626267 (Custom kit)	0.5 (stock 0.2 mg/ml in DMSO)
Lipophilic Cationic Dye (LCD)	-	BD Biosciences	-	626267 (Custom kit)	0.5 (stock 0.4 mM in DMSO)
CD45	APC-H7	BD Biosciences	2D1	560178	2 µl
CD126	V510	BD Biosciences	OKT4	740137	0.5 µl
CD171	BV421	BD Biosciences	5G3	565732	1.5 µl
Keys: R-phycoerythrin (PE); PE-Cyanine 7 (Cy7), Allophycocyanin-Hilite®7 (APC-H7), Brilliant Violet (BV). Becton Dickinson (BD) Biosciences (San Jose, CA, USA); Sigma-Aldrich (Saint Louis, Missouri, USA).					

Figure S1: Western blot of *Matrix Metalloproteinase-9 (MMP9)* expression in six patients at different time of treatment with *rh-NGF*. Multiple exposure time points, as well as the full-length blots are shown in Figure S2 and S3.

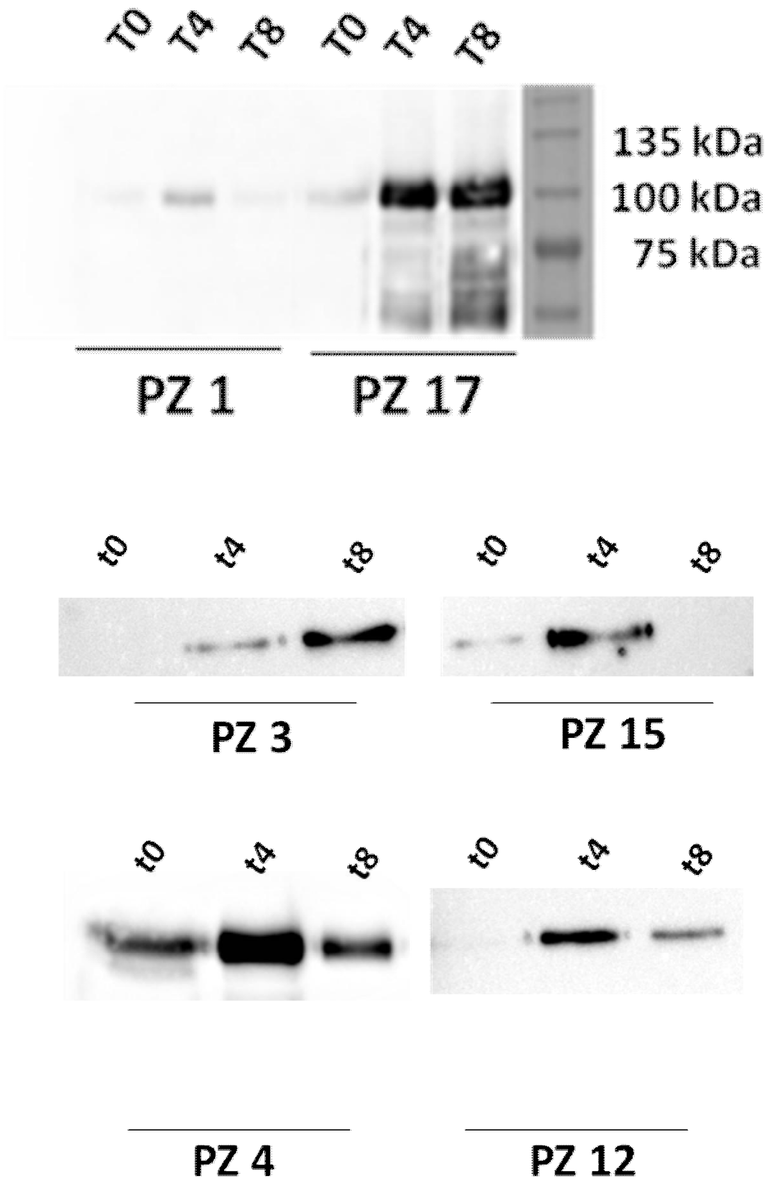

Figure S2: Full-length blot of *Matrix metalloproteinase-9 (MMP-9)* expression in two representative (pz 1 and 17) NK patients at T0, T4 and T8 timepoint during therapy (15ug of total proteins). Western Blot also shows protein molecular weights marker (Marker), a negative control (only medium) and a positive control (Sur neurons) for *MMP-9* expression (1 minute of exposure). The portion of the image discussed in Figure 3 Panel C of the main text is represented inside the rectangle. Results were obtained using a digital imaging system Alliance 4.7 (UVITEC, Cambridge, UK).

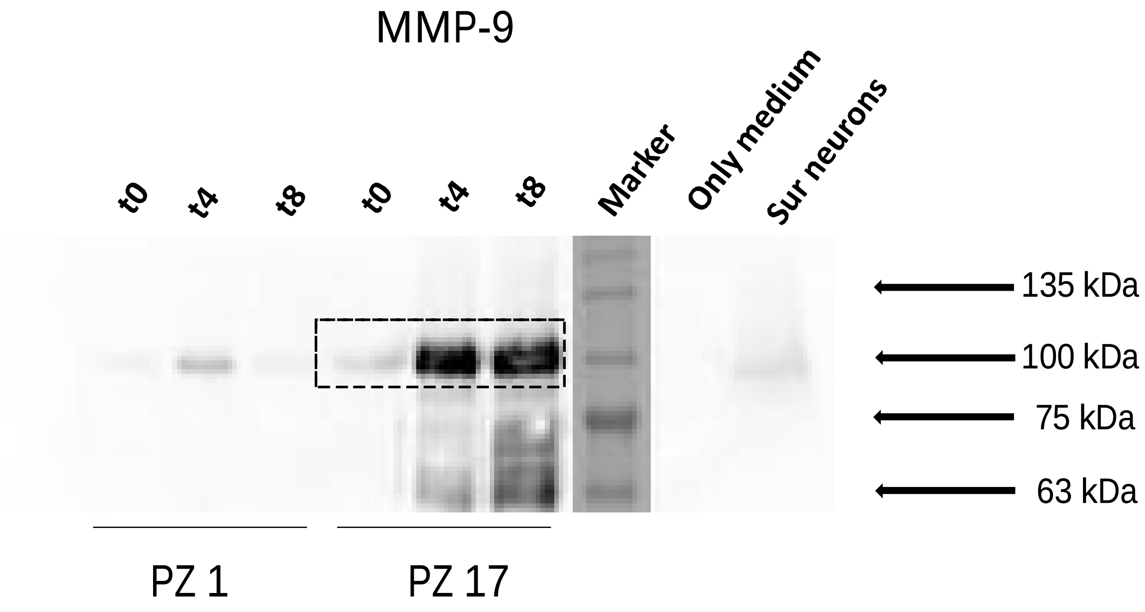

Figure S3: Panel A Full-length blot of *Matrix metalloproteinase-9 (MMP-9)* expression for patients3-4-6-9-12-15 at T0, T4 and T8 timepoint during therapy (1 minute of exposure). **Panel B** Full-length blot of *Matrix metalloproteinase-9 (MMP-9)* expression (10 minutes of exposure) used to crop the image of patient 15. **Panel C** Full-length blot of *Matrix metalloproteinase-9 (MMP-9)* expression(5 seconds of exposure) used to crop the image of patient 4. **Panel D** Full-length blot of *Matrix metalloproteinase-9 (MMP-9)* expression(1minute of exposure) used to crop the image of patients3 and 12.The portions of the image discussed in Figure S2are represented inside the rectangles. Results were obtained using a digital imaging system Alliance 4.7 (UVITEC, Cambridge, UK).Results of patients 6 and 9 were excluded from all processing as they showed anomalous data and no experimental repetitions were possible.

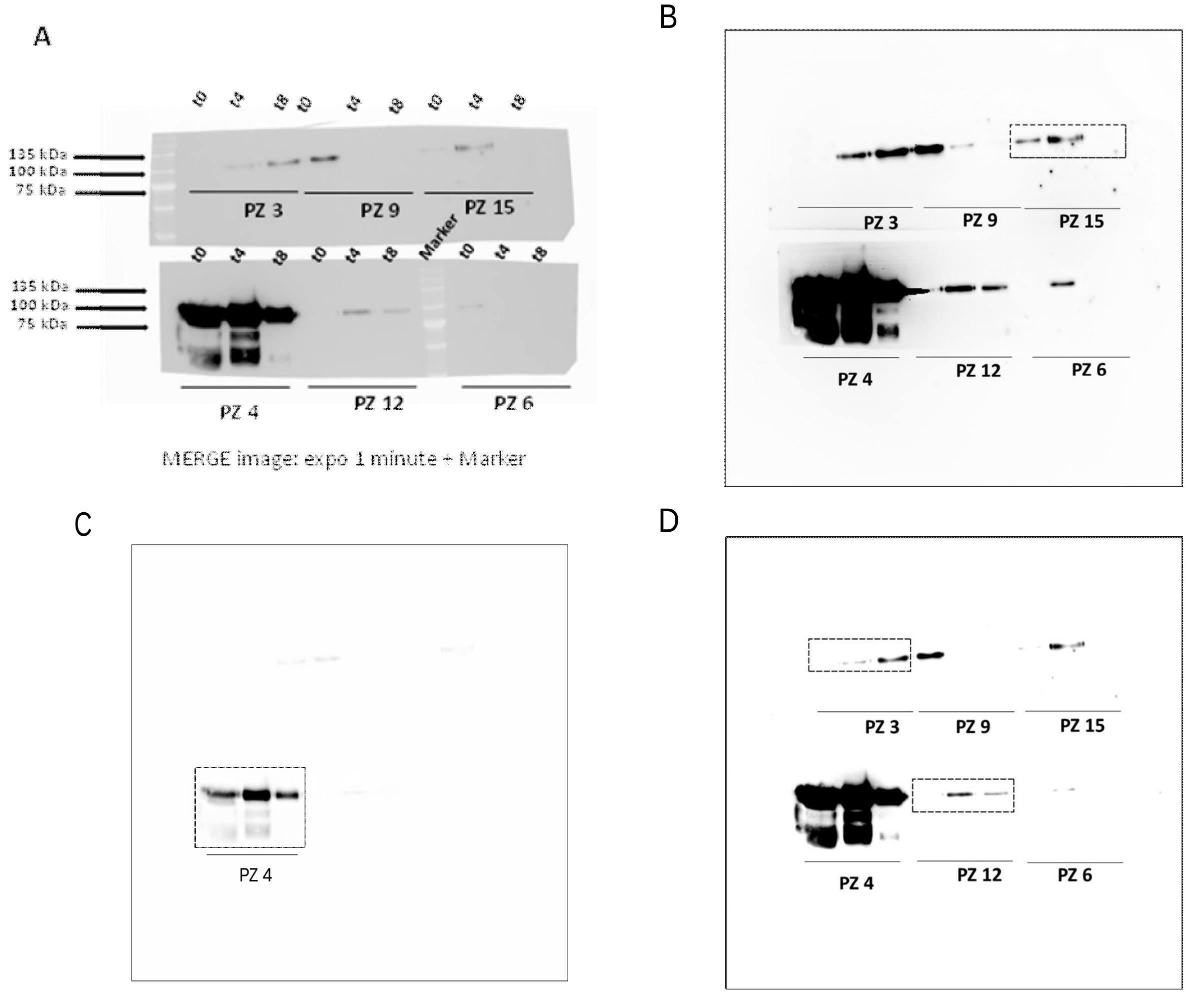

Figure S4: Rank correlation of EVs count. Spearman's coefficient of Rank correlation (rho) = 0.54 (p-value = 0.0068) between double positive EVsCD171+/CD126+ with EVs CD45+ in tears samples from patient collected before (T0) and after 4 (T4) and 8 (T8) weeks of treatment with *rhNGF*. The correlation was more driven by treated samples (T4 and T8, red and blue dots) than the naïve ones (T0, black dots), indicating the role of *rhNGF* in double positive EVs release and function.

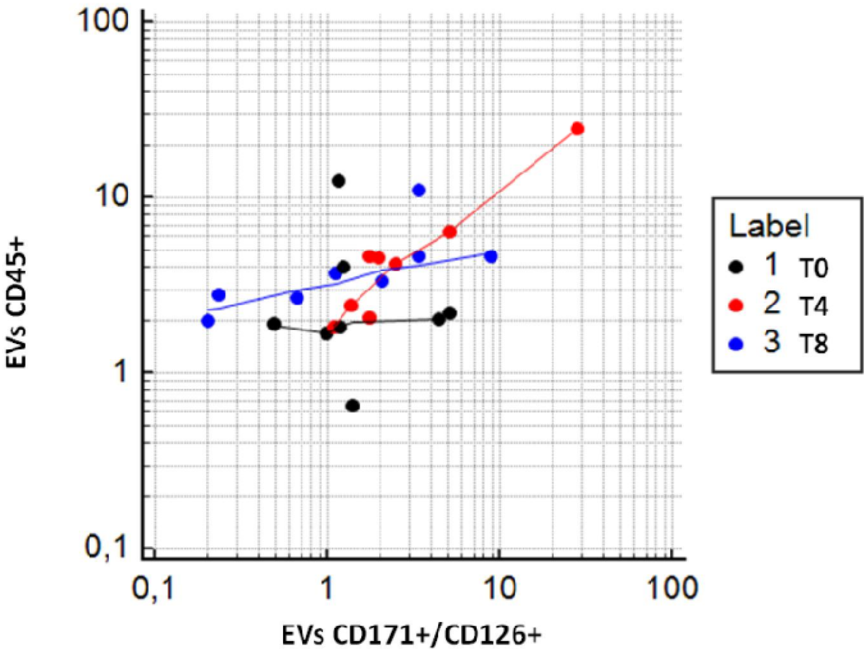

Figure S5: correlation of proteins expression in the analyzed comparisons. For both comparisons' protein expression is reported as density value of iBAQ: data points with the highest density are light blue, on the contrary data points with lowest density are bright green. The color gradient is report in the legend. While Panel A shows the correlation between protein expression in pooled tears at baseline (T0) and at 4-weeks follow up period (T4), Panel B points out the same correlation between T0 and at 8-weeks follow up period (T8).

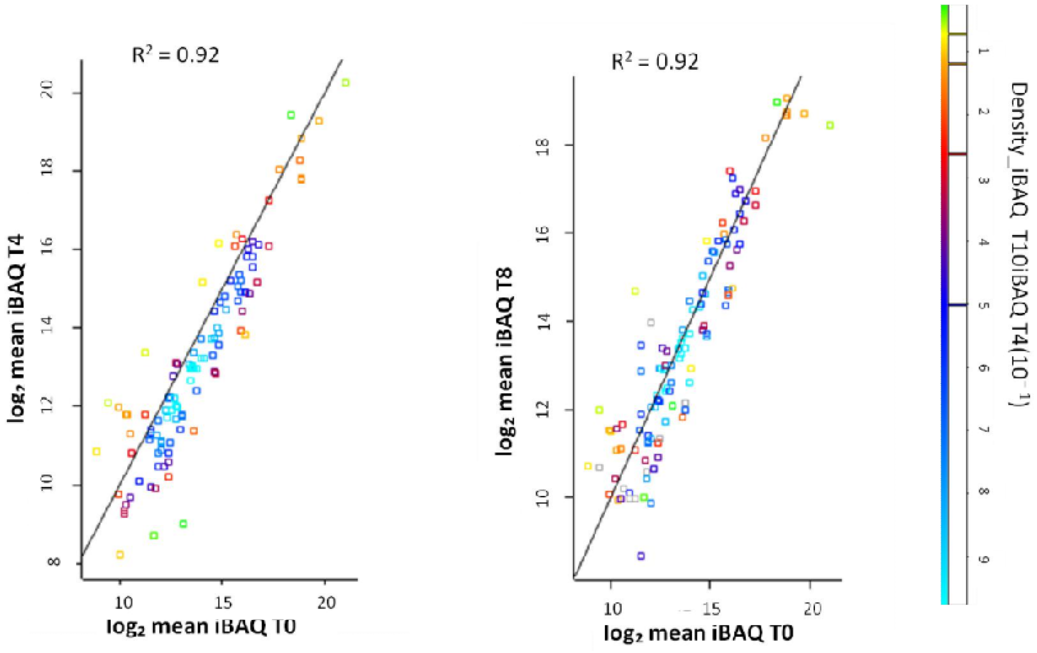

Supplement: Supplementary file 1 — Supplementary Information. [file 41598_2022_5229_MOESM1_ESM.pdf]
